# Obituary.—Dr. E. P. Gray

**Published:** 1872-08

**Authors:** 


Obituary.—Died, at St. Joseph, Mo., on the 9th inst., of congestion of the brain, Dr. E. P. Gray, formerly of this city. Dr. Gray was born in Evans, Erie County, in 1824; graduated in the Buffalo Medical College in 1849. He was Surgeon of the 78th N. Y., Regiment.

At a meeting of the St. Joseph Medical Society, the following resolutions were passed:

*Resolved*, That the members of the St. Joseph Medical Society have received the announcement of the death of Dr. E. P. Gray, for many years a member of our Society, with profound regret.

*Resolved*, That in the death of Dr. E. P. Gray, this Society suffers no common loss. Eminently qualified by a thorough education, and many years of assiduous application to his profession, he ably combatted the many diseases of suffering humanity.

*Resolved*, That in addition to his high professional qualification, his many Christian virtues, his uniform courtesy and honorable intercourse with his associates, have endeared him not only to his professional bretheen, but a large circle of private friends.

*Resolved*, That this Society deeply sympathize with his family in their sad bereavement.

*Resolved*, That this Society will attend the funeral of the deceased and wear the usual badge of mourning on the occasion.

*Resolved*, That a copy of the resolutions be published in the daily papers and a copy be furnished the family of the deceased:

Dr. H. Trevor, *President*.

Dr. T. H. Doyle, *Secretary*.

